# Evaluating the antibiotic spectrum index in a stewardship-focused clinical trial for childhood pneumonia

**DOI:** 10.1017/ice.2025.10208

**Published:** 2025-08

**Authors:** Sabrina E. Carro, Nicolas Gargurevich, Mert Sekmen, Srinivasan Suresh, Judith M. Martin, Derek J. Williams

**Affiliations:** 1 Division of Hospital Medicine, Department of Pediatrics, Vanderbilt University School of Medicine and the Monroe Carell Jr Children’s Hospital at Vanderbilt University Medical Center, Nashville, TN, USA; 2 Department of Biostatistics, Vanderbilt University Medical Center, Nashville, TN, USA; 3 Divisions of Emergency Medicine and Health Informatics, Department of Pediatrics, University of Pittsburgh School of Medicine and UPMC Children’s Hospital of Pittsburgh, Pittsburgh, PA, USA; 4 Division of General Pediatrics, Department of Pediatrics, University of Pittsburgh School of Medicine and UPMC Children’s Hospital of Pittsburgh, Pittsburgh, PA, USA

## Abstract

**Objective:**

The antibiotic spectrum index (ASI) outcome quantifies antibiotic exposure based on spectrum of activity. Our objective was to examine ASI as an exploratory outcome in the context of a recent stewardship-focused, clinical trial in childhood pneumonia that originally used a binary guideline-concordant outcome.

**Design:**

Secondary analysis of a randomized clinical trial.

**Setting:**

Two tertiary pediatric hospitals.

**Methods:**

Encounters were randomly assigned to clinical decision support (CDS) or usual care treatment arm. The ASI was calculated by summing daily ASI scores for each unique antibiotic administered. It was evaluated as a continuous and ordinal measure: No Antibiotics (ASI = 0), Narrow (1-2), Intermediate (3-4), Broad (5-7), and Very Broad (≥8). Proportional odds regression modeled the ordinal ASI outcome in the first 24 hours by treatment arm and compared to the guideline-concordance outcome. Results were stratified by emergency department (ED) disposition. We also conducted a longitudinal, descriptive analysis of day-to-day ASI for those with in-hospital dispositions.

**Results:**

We included 1027 encounters, 549 (53%) were randomized to CDS and 478 (47%) usual care respectively. ASI Category did not differ by treatment arm overall (Odds Ratio: 0.88[95% Confidence Interval: 0.70,1.09]), which mirrored binary guideline-concordance. Mean ASI was lower for concordant encounters (2.1 vs 8.4, *P* < 0.001) and across all ED dispositions. In the longitudinal analysis, there were 1137 day-to-day ASI comparisons, with only 7% representing spectrum escalations.

**Conclusions:**

The ASI outcome yielded similar results to a dichotomous concordance outcome. However, ASI provided more granular insights into antibiotic prescribing, suggesting ASI may be a useful outcome measure in future stewardship-focused trials.

## Introduction

Acute respiratory illness is the most common reason children receive antibiotics overall, and pneumonia, specifically, is responsible for more days of antibiotic use in U.S. children’s hospitals than any other condition.^1–6^ Despite national guidelines for pneumonia recommending narrow-spectrum amoxicillin for most children, broader-spectrum antibiotics are often prescribed.^7,8^ Pneumonia is an important target for initiatives promoting appropriate and judicious antibiotic use. A variety of interventions, including local practice guidelines, clinical decision support (CDS), care pathways, and formal antimicrobial stewardship programs and other activities have been studied with varying success.^9–12^ These studies most often report stewardship outcome measures such as guideline-concordance or days of therapy (DOT) per 1000 patient days.^10–12^ While useful, these measures may fail to capture important differences in the stewardship context.^13^

The Antibiotic Spectrum Index (ASI) was developed specifically to evaluate antibiotic use through a stewardship lens.^14^ The ASI quantifies antibiotic exposure based on spectrum of activity against clinically important bacterial pathogens (scores ranging from 1–13 for each antibiotic). In their index publication, Gerber and colleagues demonstrated how ASI detected differences in prescribing resulting from a new stewardship intervention that was otherwise missed by DOT per 1000 patient days.^14^ To date, several constructs of the ASI have been applied in neonatal, pediatric, and adult populations, largely in retrospective studies.^10,15,16^ To our knowledge, the ASI has not been evaluated in the context of a clinical trial as a potential outcome measure.

The Improving Care for Community-Acquired Pneumonia (ICECAP) Antibiotic CDS Trial was a two-site pragmatic randomized, usual care-controlled clinical trial that tested the effectiveness of CDS for improving guideline-concordant antibiotic prescribing in children of all ages presenting to the emergency department (ED) with pneumonia.^11^ Our objective was to examine the ASI as an exploratory outcome measure using data previously collected from the ICECAP trial.

## Methods

### Study population

The study population included all enrolled participants from the ICECAP Antibiotic CDS Trial. The study enrolled 1027 ED-based encounters for children 6 months to <18 years of age across two U.S. children’s hospitals from December 2018 to September 2020. Encounters were randomized in 4-week blocks to either antibiotic CDS plus usual care or usual care only. The intervention provided evidence-based antibiotic recommendations in alignment with national consensus guidelines on the management of childhood pneumonia.^8^ Briefly, the CDS intervention emphasized use of a narrow-spectrum beta-lactam (eg, amoxicillin or ampicillin) except in cases of severe local or systemic complications (eg, large parapneumonic effusion, respiratory failure, sepsis) or special populations (eg, treatment failure, aspiration risk, drug allergies). The trial’s primary outcome was a binary measure of exclusive guideline-concordant antibiotic prescribing during the first 24 hours of care. Those not receiving any antibiotics were classified as concordant because it was considered very unlikely that antibiotics would be withheld in children with high suspicion for bacterial disease.^6,8^ Further methodological details may be found in the trial’s primary report.^11^

### Antibiotic spectrum index

For our investigation, a daily ASI score was calculated by summating ASI scores for each unique antibiotic administered in a day, defined using consecutive 24-hour periods starting from the time of triage through either discharge from the ED, hospital, or following the 5^th^ completed hospital day. ASI scores were censored at discharge from the ED or hospital since outpatient prescribing details were not captured in the ICECAP trial. Each unique antibiotic was counted only once per day. For example, if a patient received 4 doses of ampicillin (ASI 2) and one dose of azithromycin (ASI 4) in a 24-hour period, the total ASI would be 6 for that period. Non-systemic antibiotics, such as topical, otic, ophthalmic, inhalation, or nebulization, were excluded from ASI calculations. Rifaximin and enteral erythromycin, which are used most often in the management of gastrointestinal disorders and not routinely prescribed for acute pneumonia, were also excluded.^17^

ASI was evaluated both as a continuous measure and as an ordinal construct. For the latter, daily ASI scores were organized into 5 mutually exclusive, ranked categories: No Antibiotics (ASI = 0), Narrow (1-2), Intermediate (3-4), Broad (5-7), and Very Broad (≥8). The cut points were defined to create clinically relevant categories in the specific context of childhood pneumonia (Table 1). For example, Narrow corresponds to treatment with amoxicillin or ampicillin monotherapy (ASI 2), the recommended first-line antibiotic for uncomplicated bacterial pneumonia, while Very Broad corresponds to treatment strategies for complex or very severe disease (eg, ceftriaxone plus vancomycin; ASI = 10). To leverage the full cohort of ED and in-hospital encounters, our primary analyses focused on the ASI during the first 24 hours to align with the trial’s primary concordance outcome.

**Table 1.** ASI scores and category designations. Included is an abbreviated summary of antibiotics with respective ASI scores in parenthesis. The full list can be found in the index publication.^14^ The ASI was modified to include Nafcillin which was assigned a score (ASI 1) in alignment with the two previously reported penicillin agents as noted in the table. Abbreviations: ASI: antibiotic spectrum index

ASI Category (ASI Score Range)	Antibiotic Examples (ASI Score)
No antibiotics (0)	N/A
Narrow (1-2)	Oxacillin/Dicloxacillin (1)
	Ampicillin/Amoxicillin (2)
Intermediate (3-4)	1st Generation Cephalosporins (3)
	Most 2nd Generation Cephalosporins, Some 3rd Generation (4)
	Clindamycin (4)
	Azithromycin (4)
Broad (5-7)	Ceftriaxone (5)
	Vancomycin (5)
	Most 3rd Generation Cephalosporins, Some 2nd Generation (5)
	Ampicillin-Sulbactam/ Amoxicillin- Clavulanate (6)
	Cefepime and 4th Generation Cephalosporins (6)
	Ampicillin (2) + Azithromycin (4) = (6)
Very Broad (≥8)	Ceftaroline (8)
	Levofloxacin (9)
	Ceftriaxone (5) + Vancomycin (5) = (10)
	Ceftriaxone (5) + Vancomycin (5) + Azithromycin (4) = (14)

Additionally, we used the ASI to explore day-to-day changes in antibiotic prescribing in the hospital setting using in-hospital encounters with initial ED dispositions of either Acute Care (referred herein as Inpatient) or Intensive Care (ICU). For this, we calculated the difference in categorical ASI between consecutive hospital days for each encounter (eg, comparing hospital day 2 to hospital day 1, hospital day 3 to hospital day 2). Discharge day was censored due to incomplete prescribing data. These differences, reported as Delta ASI (∆ ASI), yield positive values when antibiotic spectrum is escalated (+1 per category change), negative values when de-escalated (-1 per category change), and a zero value when there is no change in the ASI category.

### Analyses

To approximate the trial’s primary analysis of guideline concordance (estimated using unadjusted logistic regression), we fit an unadjusted proportional odds logistic regression model with treatment arm as the exposure (odds ratios >1 indicate *increased* odds of receiving *more* narrow spectrum therapy). Though we did not expect the effect estimates to align given the differing outcome constructs, we anticipated the direction of observed associations would be aligned since increasing narrow-spectrum antibiotic treatment correlates with more guideline-concordant prescribing in the context of pediatric pneumonia.

To further explore the ASI outcome constructs, we also summarized ASI data by guideline concordance and ED Disposition (Outpatient, Inpatient, ICU), using Welsh’s T-Test to compare mean ASI scores across groups. A Sankey Diagram, which is a data visualization technique that emphasizes changes in state over time, was generated to visualize day-to-day changes in ASI category. All analyses were conducted using R version 4.3.2.

## Results

### Study population

All 1027 ICECAP encounters (median age 4.2 yr) included in the primary analysis were retained for this evaluation, including 478 (47%) randomized to usual care and 549 (53%) to antibiotic CDS. In total, 431 (42%) encounters were discharged home from the ED, 404 (39%) were admitted to inpatient acute care, and 192 (19%) were admitted to intensive care (Table 2). Approximately half of all encounters were concordant with guideline-recommended antibiotic use during the first 24 hours of care (Supplement 1).

**Table 2.** Patient population characteristics. Categorical data presented as n (% frequency) and continuous data as median (interquartile range). For the improving care for community-acquired pneumonia (ICECAP) antibiotic clinical decision support trial characteristics, please refer to the original publication.^11^ ICU: Intensive Care Unit. IQR: interquartile range

Characteristic	Emergency department disposition			
	Overall (n = 1027)	Outpatient (n = 431)	Inpatient (n = 404)	ICU (n = 192)
Age in years [Median (IQR)]	4.2 (1.9, 8.4)	4.9 (2.3, 8.6)	4.0 (1.9, 7.4)	3.5 (1.4, 8.6)
Sex [n (%)]				
Male	564 (54.9)	239 (55.5)	215 (53.2)	110 (57.3)
Female	463 (45.1)	192 (44.5)	189 (46.8)	82 (42.7)
Race [n (%)]				
White	693 (67.5)	284 (65.9)	274 (67.8)	135 (70.3)
Black	196 (19.1)	93 (21.6)	69 (17.1)	34 (17.7)
Asian	31 (3.02)	13 (3.02)	11 (2.72)	7 (3.65)
Mixed	22 (2.14)	10 (2.32)	8 (1.98)	4 (2.08)
Other	44 (4.28)	17 (3.94)	21 (5.20)	6 (3.13)
Unknown	41 (3.99)	14 (3.25)	21 (5.20)	6 (3.13)
Ethnicity [n (%)]				
Hispanic	96 (9.35)	44 (10.2)	36 (8.91)	16 (8.33)
Not Hispanic	862 (83.9)	372 (86.3)	334 (82.7)	156 (81.3)
Unknown	69 (6.72)	15 (3.48)	34 (8.42)	20 (10.4)
Comorbidity [n (%)]				
Non-Chronic	641 (62.4)	336 (78)	224 (55.4)	81 (42.2)
Non-Complex Chronic	131 (12.8)	44 (10.2)	65 (16.1)	22 (11.5)
Complex Chronic	255 (24.8)	51 (11.8)	115 (28.5)	89 (46.4)
Insurance [n (%)]				
Public	561 (54.6)	233 (54.1)	219 (54.2)	109 (56.8)
Private	422 (41.1)	182 (42.2)	167 (41.3)	73 (38.0)
Self-Pay	41 (3.99)	16 (3.71)	15 (3.71)	10 (5.21)
Unknown	3 (0.29)	0 (0.00)	3 (0.74)	0 (0.00)
Enrolling site [n (%)]				
Site 1	492 (47.9)	186 (43.2)	191 (47.3)	115 (59.9)
Site 2	535 (52.1)	245 (56.8)	213 (52.7)	77 (40.1)

During this period, 441 (43%) encounters either received no antibiotics or received amoxicillin or ampicillin (ASI of 2 or less). These treatment strategies were very common among those discharged from the ED (70%) but constituted a minority of inpatient (30%) and ICU (10%) encounters. Accordingly, mean ASI increased as the level of care escalated, corresponding to Narrow for those discharged home from the ED, and Broad and Very Broad for those admitted to inpatient acute care and intensive care, respectively.

### ASI by treatment arm

Consistent with the trial’s outcome of guideline concordance at 24 hours, ASI did not differ by treatment arm overall (OR: 0.88 [95% CI: 0.70, 1.09]). ASI did not differ by ED Disposition: Outpatient (1.10 [0.78, 1.55]), Inpatient (0.93 [0.66, 1.32]), or ICU disposition (0.96 [0.54, 1.71]) (Table 3). For outpatient encounters, there was more narrow antibiotic use than no antibiotics in the CDS than the usual care arm (Supplement 2). During this period, the mean ASI was significantly lower for concordant vs discordant encounters (2.1 vs 8.4, p-value < 0.001), corresponding to Narrow and Very Broad categories, respectively. This pattern was observed across each subgroup defined by ED Disposition (Table 4). When evaluating only encounters that received antibiotics in a sensitivity analysis, this pattern persisted (Supplement 3).

**Table 3.** Comparison of unadjusted odds ratios (ORs) Against ICECAP Trial. The unadjusted ORs in the ICECAP trial primary outcome and daily ASI Category in the first 24 hours of care are shown. For the ICECAP outcome, an OR > 1 reflects increased odds of being guideline concordant. For the ASI category outcome, an OR > 1 demonstrates increased odds of being in a lower—and narrower—ASI category. Abbreviations: OR: odds ratio. CI: confidence interval. ICECAP: improving care for community-acquired pneumonia. asi: antibiotic spectrum index. ED: emergency department. ICU: intensive care unit

	ICECAP Odds Ratio (95% CI)	ASI Category Odds Ratio (95% CI)
Overall	0.94 (0.73, 1.20)	0.88 (0.70, 1.09)
**ED Disposition**		
Outpatient	1.53 (1.01, 2.33)	1.10 (0.78, 1.55)
Inpatient	0.88 (0.60, 1.30)	0.93 (0.66, 1.32)
ICU	0.56 (0.28, 1.11)	0.96 (0.54, 1.71)

**Table 4.** Comparison of mean ASI between those considered to be guideline-concordant versus discordant in all encounters. Abbreviations ASI: antibiotic spectrum index. ED: emergency department. ICU: intensive care unit

	Guideline-Concordance Mean ASI (Standard Deviation)		
	Concordant (n = 543)	Discordant (n = 484)	*p*-value
Overall	2.1 (2.8)	8.4 (4.5)	<0.001
**ED Disposition**			
Outpatient (n = 431)	1.0 (1.1)	5.4 (1.9)	<0.001
Inpatient (n = 404)	3.0 (2.6)	8.7 (4.2)	<0.001
ICU (n = 192)	5.8 (6.0)	10.6 (5.1)	<0.001

### Inpatient longitudinal ASI

**Figure 1.**
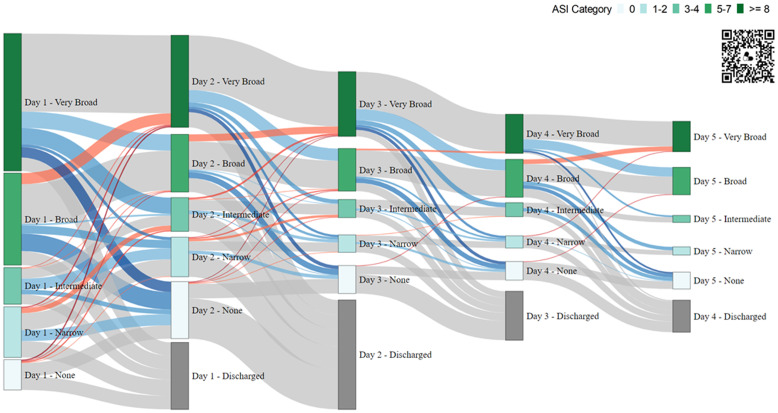
Antibiotic spectrum index (ASI) Category Across Hospitalization Days. This Sankey Diagram displays day-to-day changes in ASI Category for all in-hospital encounters through discharge. Each vertical column represents a hospital day, with nodes (vertical bars) representing encounters in each ASI Category (No Antibiotics, Narrow, Intermediate, Broad, Very Broad) and horizontal wavy lines representing the flow, or change between days. *Note*: The QR code links to an interactive HTML version of the diagram with additional embedded data. Node Height: Proportional to number of encounters in that ASI category. Node Color: Corresponds to associated ASI Category (see Key in diagram); nodes in gray appear on Days 2-5 to account for encounters discharged the prior day. Flows (Connections) Between Columns of Nodes: Changes in ASI Category day-to-day, with the flow width proportional to the number of encounters undergoing that change. Flow Color: Changes (∆) in ASI Category, thus antibiotic spectrum, quantified as escalation (red), de-escalation (blue), and no change (gray); intensity of shading is proportional to magnitude of antibiotic spectrum change. ASI, antibiotic spectrum index.

Of the 596 in-hospital encounters, 481 (80.1%) remained in the hospital through day 2; and 154 (25.8%) remained in the hospital through day 5. Over the course of 5 days, 244 (40.9%) of the 596 included encounters demonstrated an antibiotic spectrum de-escalation event, 41 (6.1%) experienced an escalation, and 107 (18.0%) had both escalation and de-escalation events. A Sankey diagram visualized clinically relevant changes in ASI by hospital day at a more granular scale (Figure 1). Overall, 1137 discrete day-to-day antibiotic spectrum events were evaluated. Most (63.9%) corresponded to no change in ASI, about one-third (29.3%) were classified as de-escalation events, and few (7.0%) were classified as escalation events.

**Figure 2.**
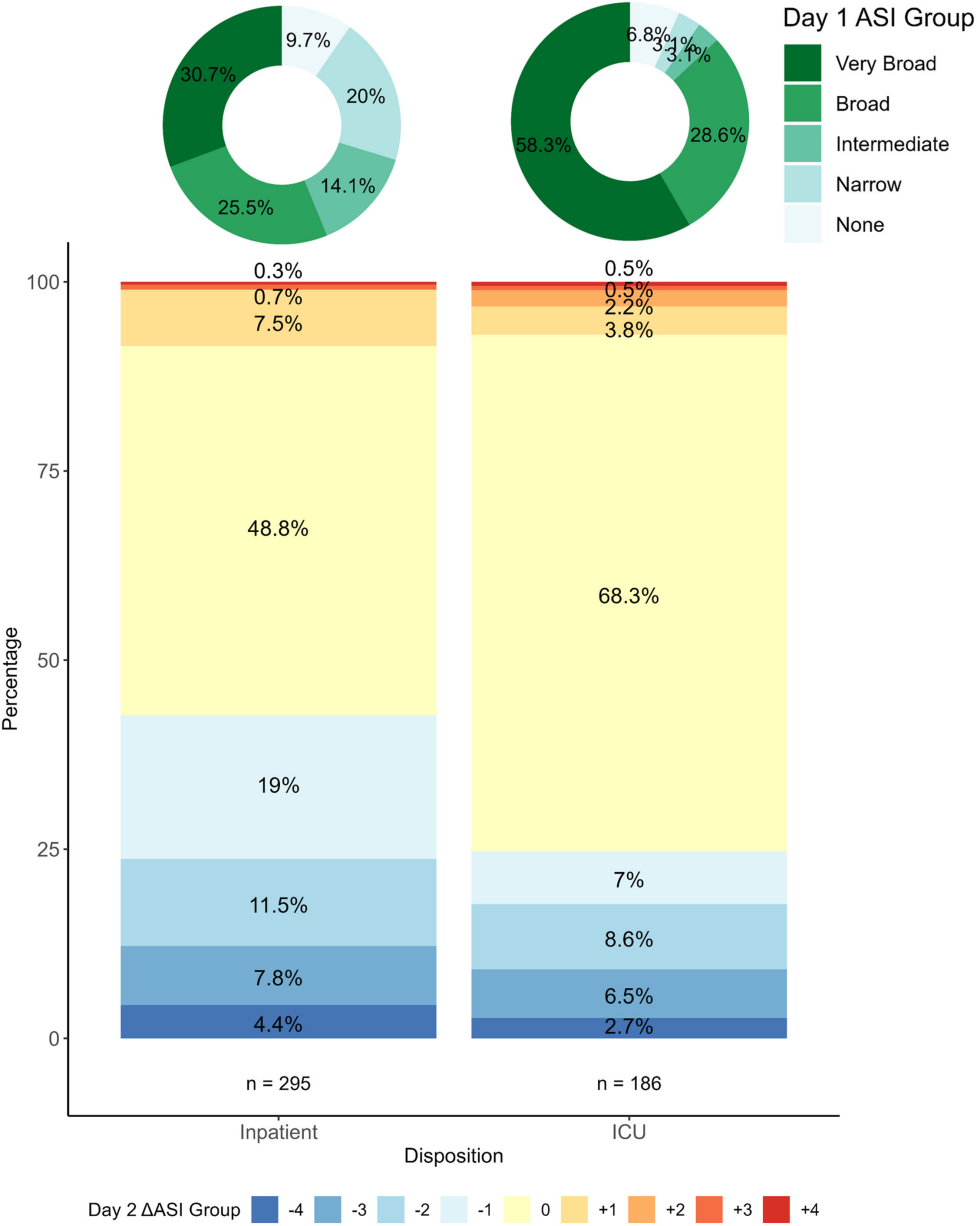
Change in ASI Category from Day 1 to Day 2 by Hospital Disposition. Donut charts on the top of the figure show the distribution of ASI category on Day 1 for Inpatient and ICU encounters. The bar plot below shows the change in ASI category from Day 1 to Day 2. The change is zero if the ASI category on Day 2 was the same as Day 1. Positive or negative values indicate escalations (red) or de-escalations (blue) respectively; a value of +1 represents an escalation up one ASI category whereas -1 is a de-escalation down one ASI category. ASI, antibiotic spectrum index; ICU, intensive care unit.

Focusing on ASI changes by disposition, 227 (56.2%) Inpatient and 167 (87.0%) ICU encounters were classified as Broad or Very Broad on day 1. On day 2, 126 (42.7%) inpatient encounters and 46 (24.7%) ICU encounters were de-escalated, whereas escalations were noted in <10% of encounters in both groups (Figure 2). Both de-escalation and escalation events were less common on subsequent hospital days.

## Discussion

Application of the ASI as an ordinal construct in a prior stewardship-focused clinical trial demonstrated similar overall results to the trial’s primary dichotomous outcome, while also providing a more nuanced, semi-quantitative view of antibiotic use. The ASI is a potentially useful outcome measure for future clinical trials and antibiotic stewardship interventions.

Importantly, the ASI provided evidence of intervention effects that were obscured by the trial’s concordance outcome. An example of this, we noted an increased proportion of narrow-spectrum antibiotic use in outpatient encounters compared to no antibiotic use, a difference only revealed after applying the ASI. This is attributed to the construction of our ordinal categories where we opted to include no antibiotics as its own treatment strategy opposed to guideline-concordance. Although guideline-concordant treatment for pneumonia includes no antibiotics as well as narrow spectrum, it does not inherently distinguish the difference between these two strategies.

An advantage of the ASI is its ability to quantify antibiotic exposure by spectrum within a single, ordinal, or continuous measure. For example, among guideline-concordant encounters in the trial, ASI captured important differences in prescribing by ED disposition, illustrating that outpatients most often received narrow-spectrum therapy or no antibiotics while those in the ICU were most often broad spectrum, despite all being considered guideline-concordant. Similarly, comparing ASI between concordant and discordant encounters provided insights not apparent in the concordance outcome. The ASI may be particularly useful, then, for measuring antibiotic use for specific conditions when treatment strategies are expected to vary due to differences across levels of care, institutions, or patient sub-populations.

The ASI appears advantageous when employed to evaluate a specific antibiotic indication in a stewardship context. This score lends itself well specifically to pneumonia because most cases of pneumonia can be safely treated with a beta-lactam (ASI 2) when the etiology is presumed to be bacterial or no antibiotics (ASI 0) when it is presumed to be viral. Only under certain scenarios would broader antibiotics be recommended such as ceftriaxone (ASI 5) or ampicillin-sulbactam (ASI 6). The ASI could be applied to other infectious etiologies with the first line treatment strategy favors narrower spectrum use such as acute otitis media, skin and soft tissue infections, and UTIs.

Another strength of the ASI is the potential to examine temporal changes in antibiotic use. Though the ICECAP antibiotic trial was not designed to evaluate longitudinal patterns of ASI during hospitalization (post-ED decision support was not available), our exploratory analyses support the hypothesis of treatment inertia in the hospital setting, namely that initial antibiotic treatment often predicts ongoing treatment course. For example, broad or very broad-spectrum antibiotic therapies were the most common empiric (day 1) treatment strategies for both inpatient and ICU encounters. This initial strategy was unchanged for half of inpatients and over two-thirds of the ICU encounters on day 2. Across all change events evaluated through day 5, most did not alter ASI category and less than 10% were escalation events. Limitations of the current dataset notwithstanding, robust longitudinal assessments using the ASI, capturing both index encounter and discharge prescribing data (including duration), provides a useful mechanistic outcome for measuring encounter-level changes in antibiotic initiation, spectrum, and duration in a single measure. This is therefore an attractive metric for evaluating multifaceted or longitudinal stewardship interventions and other clinical innovations.

The ASI has been explored by others in various retrospective applications.^16,18,19^ Plattner et al used the ASI to quantify patient-level antibiotic changes in critically ill children with pneumonia following implementation of a rapid multiplex PCR panel,^16^ while Bittmann et al. compared ASI scores on discrete days to look at pathogen-specific trends in patient-level antibiotic use for hospital and ventilator acquired pneumonia in the adult ICU.^19^ Both of these studies were retrospective and neither evaluated ASI as a single, continuous measure nor duration of therapy. However, the ASI in both cases allowed the study teams to reliably quantify antibiotic exposure across varied treatment settings and provide foundational examples for future work.

While the ASI has many features that make it a useful outcome measure, it has limitations, including sometimes equating two antibiotic choices, such as ceftriaxone (ASI 5) and vancomycin (ASI 5), that in clinical practice are viewed quite differently, both for their indications as well as importance in the setting of antimicrobial resistance. Additionally, ASI scores do not account for local resistance patterns. For example, in areas with high rates of penicillin resistance, ceftriaxone (ASI 5) may be recommended over amoxicillin or ampicillin (ASI 2), making direct comparisons challenging.^8,20^ Similarly, the ASI score and how it changes do not always equate with appropriateness. Outcomes to evaluate the clinical context and measure effectiveness, then, are important to complement studies that employ the ASI.

In the first description of the ASI and its potential utility, Gerber et al. used a large administrative dataset to compare ASI per antibiotic day against DOT per 1000 patient days in the context of a targeted ASP intervention for pediatric pneumonia. Their ASI construct outcome demonstrated important differences in antibiotic prescribing patterns not detected by the common DOT measure. The ASI has since been applied in different ways (eg, ASI per 1000 patient days or as a discrete daily measure) alongside other common stewardship metrics.^10,15,16,21^ It has also proven advantageous in characterizing variation in antibiotic use between hospitals and populations.^14,22–24^ Thoughtful selection of the right ASI constructs, including consideration of the clinical context, and measurement of safety or clinical endpoints (such as clinical care escalation) are important to ensure meaningful conclusions.

Limitations of our analyses stem primarily from the secondary, post-hoc nature of the study design. For example, the trial did not collect discharge prescriptions or post-encounter data. Given the short hospital stays for most patients, discharge censoring resulted in important information loss. Similarly, we were not able to correlate antibiotic changes with clinical status. Thus, the appropriateness of escalation and de-escalation events was impossible to evaluate. Lastly, robust health-related social needs data were not collected, limiting our ability to apply the ASI to detect differences in antibiotic prescribing amongst subpopulations defined by these characteristics. Thoughtfully designed studies can overcome these limitations.

We evaluated both continuous and ordinal constructs of the ASI in the context of a previously conducted stewardship-focused clinical trial. We re-demonstrated the utility of the ASI for pediatric pneumonia studies and provided examples of how the ASI could provide deeper insights into antibiotic prescribing variation and use patterns, including longitudinal trends and response to interventions. We conclude that the ASI is a useful and flexible stewardship measure, but one that should ideally be combined with measures of clinical importance when evaluating impact.

## Supporting information

Carro et al. supplementary material 1 Carro et al. supplementary material

Carro et al. supplementary material 2 Carro et al. supplementary material

## Supplementary material

The supplementary material for this article can be found at https://doi.org/10.1017/ice.2025.10208
