# Supplementary material for: Evaluating the antibiotic spectrum index in a stewardship-focused clinical trial for childhood pneumonia
Source: Infect Control Hosp Epidemiol. 2025 Jun 23;46(8):805–11. doi: 10.1017/ice.2025.10208 (PMC12483619; doi:10.1017/ice.2025.10208)
Supplement: Carro et al. supplementary material 1 — Carro et al. supplementary material [file S0899823X25102080sup001.docx]

|  |  |  | Emergency Department Disposition | | |
| --- | --- | --- | --- | --- | --- |
|  |  | Overall  n= 1027 | Outpatient  n= 431 | Inpatient  n= 404 | ICU  n= 192 |
| Guideline Concordant [n (%)] | | |  |  |  |
|  |  |  |  |  |  |
|  | Concordant | 543 (52.9) | 304 (70.5) | 195 (48.3) | 44 (22.9) |
|  | Discordant | 484 (47.1) | 127 (29.5) | 209 (51.7) | 148 (77.1) |
| ASI Category [n (%)] | | | |  |  |
|  | No Antibiotics (0) | 202 (19.7) | 150 (34.8) | 39 (9.65) | 13 (6.77) |
|  | Narrow (1-2) | 239 (23.3) | 152 (35.3) | 81 (20.0) | 6 (3.13) |
|  | Intermediate (3-4) | 111 (10.8) | 48 (11.1) | 57 (14.1) | 6 (3.13) |
|  | Broad (5-7) | 226 (22.0) | 68 (15.8) | 103 (25.5) | 55 (28.6) |
|  | Very Broad ( >/= 8) | 249 (24.2) | 13 (3.02) | 124 (30.7) | 112 (58.3) |

Supplement 1: Summarization of antibiotic use for the first 24 hours in all enrolled encounters. All values are expressed as N (%). ICU: Intensive Care Unit. ASI: Antibiotic Spectrum Index

Supplement 2: The percentage of each ASI category in the ICECAP trial treatment arms in the first 24 hours of care in each ED Disposition. Notably, there is increased Narrow spectrum antibiotic use in the Outpatient CDS arm compared to Usual Care. ICECAP: Improving Care for Community-Acquired Pneumonia. CDS: Clinical Decision Support. ASI: Antibiotic Spectrum Index. ICU: Intensive Care Unit.

|  | Guideline-Concordance in Encounters Receiving Antibiotics  Mean ASI (Standard Deviation) | | | | |
| --- | --- | --- | --- | --- | --- |
|  | | Concordant (n=341) | | Discordant (n=484) | p-value |
| Overall | | 3.4 (2.9) | | 8.4 (4.5) | < 0.001 |
| ED Disposition | |  |  | |  |
|  | Outpatient (n=281) | 2.1 (0.4) | | 5.4 (1.9) | < 0.001 |
|  | Inpatient (n=365) | 3.7 (2.4) | | 8.7 (4.2) | < 0.001 |
|  | ICU (n=179) | 8.2 (5.7) | | 10.6 (5.1) | 0.036 |

Supplement 3: Comparison of mean ASI between those considered to be guideline-concordant versus discordant only including encounters receiving antibiotics. Abbreviations ASI: Antibiotic Spectrum Index. ED: Emergency Department. ICU: Intensive Care Unit
